# Exome-Wide Association Study of Endometrial Cancer in a Multiethnic Population

**DOI:** 10.1371/journal.pone.0097045

**Published:** 2014-05-08

**Authors:** Maxine M. Chen, Marta Crous-Bou, Veronica W. Setiawan, Jennifer Prescott, Sara H. Olson, Nicolas Wentzensen, Amanda Black, Louise Brinton, Chu Chen, Constance Chen, Linda S. Cook, Jennifer Doherty, Christine M. Friedenreich, Susan E. Hankinson, Patricia Hartge, Brian E. Henderson, David J. Hunter, Loic Le Marchand, Xiaolin Liang, Jolanta Lissowska, Lingeng Lu, Irene Orlow, Stacey Petruzella, Silvia Polidoro, Loreall Pooler, Timothy R. Rebbeck, Harvey Risch, Carlotta Sacerdote, Frederick Schumacher, Xin Sheng, Xiao-ou Shu, Noel S. Weiss, Lucy Xia, David Van Den Berg, Hannah P. Yang, Herbert Yu, Stephen Chanock, Christopher Haiman, Peter Kraft, Immaculata De Vivo

**Affiliations:** 1 Program in Genetic Epidemiology and Statistical Genetics, Harvard School of Public Health, Boston, Massachusetts, United States of America; 2 Channing Division of Network Medicine, Department of Medicine, Brigham and Women's Hospital and Harvard Medical School, Boston, Massachusetts, United States of America; 3 University of Southern California, Los Angeles, California, United States of America; 4 Memorial Sloan-Kettering Cancer Center, New York, New York, United States of America; 5 Division of Cancer Epidemiology and Genetics, National Cancer Institute, Bethesda, Maryland, United States of America; 6 Fred Hutchinson Cancer Research Center, Seattle, Washington, United States of America; 7 University of New Mexico, Albuquerque, New Mexico, United States of America; 8 Alberta Health Services – CancerControl Alberta, Calgary, Alberta, Canada; 9 Geisel School of Medicine, Dartmouth College, Hanover, New Hampshire, United States of America; 10 Department of Biostatistics and Epidemiology, University of Massachusetts, Amherst, Massachusetts, United States of America; 11 University of Hawaii Cancer Center, Honolulu, Hawaii, United States of America; 12 Department of Cancer Epidemiology and Prevention, M Sklodowska-Curie Cancer Center and Institute of Oncology, Warsaw, Poland; 13 Yale University School of Public Health, New Haven, Connecticut, United States of America; 14 Center for Cancer Prevention (CPO-Piemonte), Turin, Italy; 15 Human Genetic Foundation (HuGeF), Turin, Italy; 16 Center for Clinical Epidemiology and Biostatistics, University of Pennsylvania School of Medicine, Philadelphia, Pennsylvania, United States of America; 17 Cancer Prevention Institute of California, Fremont, California, United States of America; 18 Vanderbilt University Medical Center, Nashville, Tennessee, United States of America; 19 University of Washington, Seattle, Seattle, Washington, United States of America; IFOM, Fondazione Istituto FIRC di Oncologia Molecolare, Italy

## Abstract

Endometrial cancer (EC) contributes substantially to total burden of cancer morbidity and mortality in the United States. Family history is a known risk factor for EC, thus genetic factors may play a role in EC pathogenesis. Three previous genome-wide association studies (GWAS) have found only one locus associated with EC, suggesting that common variants with large effects may not contribute greatly to EC risk. Alternatively, we hypothesize that rare variants may contribute to EC risk. We conducted an exome-wide association study (EXWAS) of EC using the Infinium HumanExome BeadChip in order to identify rare variants associated with EC risk. We successfully genotyped 177,139 variants in a multiethnic population of 1,055 cases and 1,778 controls from four studies that were part of the Epidemiology of Endometrial Cancer Consortium (E2C2). No variants reached global significance in the study, suggesting that more power is needed to detect modest associations between rare genetic variants and risk of EC.

## Introduction

Endometrial cancer (EC), a cancer of the uterine epithelial lining that typically occurs near or after menopause, is the most common cancer of the female reproductive organs and the 10th leading cause of cancer death in women in the developed world [1]–[3]. EC is strongly associated with estrogen-only post-menopausal hormone therapy [4], [5] and excess body weight [6] due to increased aromatization of C-19 steroids by excess adipose tissue [7]. These risk factors support the “unopposed estrogen” hypothesis in which EC may develop because of the unchecked mitogenic effects of estrogen in the absence of sufficient progesterone [8]. Some studies have shown that family history increases risk two to three-fold in younger women who have a first-degree female relative with EC [9], [10], while among older women the association is less strong. In addition, there is an increased risk of EC in women with Lynch syndrome [11], a hereditary autosomal dominant condition that confers a high risk of colorectal cancer as well. These observations suggest that germline genetics may contribute to EC susceptibility.

Genome-wide association studies (GWAS) have successfully identified more than a hundred susceptibility loci for a variety of cancer types [12]. Three GWAS studies of EC have been conducted to date with only one identifying a novel genome-wide significant locus, rs4430796, (p  =  7.1 × 10^−10^) associated with EC [13] at the *HNF1B* gene region on chromosome 17q12. Two independent studies subsequently replicated the association with rs4450796 [14], [15]. However, two other GWAS studies of EC [14], [16] were not able to identify additional genome-wide significant loci, suggesting that common variants with large effects may not highly contribute to the familial risk of EC.

Most risk alleles discovered through GWAS have modest effect sizes that do not account for much heritability of common diseases [17]. Moreover, GWAS studies have focused on common variants (>5%) in the general population. Low frequency variants make up a large fraction of genetic variation in humans and may explain a substantial portion of the heritability in cancer etiology. Recent exome-sequencing studies have found rare variants in candidate susceptibility genes for familial colorectal cancer [18], breast cancer [19], and prostate cancer [20], suggesting that analysis of rare variants may also provide insight into the etiology of EC. However, exome-sequencing studies require samples sizes that are not amenable to large epidemiological studies due to the high cost currently needed to achieve sufficient statistical power.

There has been a push to develop statistically powerful, yet relatively inexpensive, methods to detect associations for rare variants with larger effect sizes. Illumina has recently developed the Infinium HumanExome BeadChip (exome array) from non-synonymous variants found at least 3 times on more than 2 data sets from the whole-exome sequencing of more than 12,000 individuals. This array provides a platform from which we can begin to survey the landscape of rare variation in a large number of samples.

We genotyped rare variants in a multiethnic population of 3,067 women (1,169 EC cases and 1,898 controls) from the Epidemiology of Endometrial Cancer Consortium (E2C2) [21] in order to test the hypothesis that rare variants in coding regions may be associated with EC risk.

## Methods

Ethics committee from each participating study (Alberta Health Services; Estrogen, Diet, Genetics and Endometrial Cancer Study; Multiethnic Cohort Study) obtained written informed consent from all study participants. All written consent was approved from the Institutional Review Board (IRB) from each institution (Alberta Health Services, Canada; Memorial Sloan Kettering, USA; University of Hawaii Cancer Center, USA; Keck School of Medicine-University of Southern California, USA).

Alberta Health Services, Memorial Sloan Kettering, University of Hawaii Cancer Center, and University of Southern California institutional review boards specifically approved the present study (Exome-Wide Association Study of Endometrial Cancer), as well as the written consent obtained from participants.

Participating studies also obtained IRB certification, permitting data sharing according to the NIH Policy for Sharing of Data Obtained in NIH Supported or Conducted Genome- Wide Association studies (GWAS).

### Study Population

Exome array genotyping was performed on 3,067 samples from 3 retrospective case-control studies: the Alberta Health Services Study (AHS) [22], the Estrogen, Diet, Genetics and Endometrial Cancer study (EDGE) [23], and the Fred Hutchinson Cancer Research Center (FHCRC) study and 1 case-control study nested within the prospective Multiethnic Cohort Study (MEC) [24]. Studies participating in this analysis are described in Table 1 and in our previous GWAS[14]. Of the women included in the study, 1,169 were EC cases and 1,898 were controls. Cases were restricted to those diagnosed with the most common subtype of EC (type I) while controls were cancer free and had an intact uterus. Controls were matched to cases by age and study site.

**Table 1:** Studies participating in the exome-wide association study of endometrial cancer.*

Study	Study Acronym	Study Design	Cases	Controls	Location	Mean BMI at diagnosis (cases)	Mean age at diagnosis (cases)	Total
Alberta Health Services	AHS	Case-Control	517	937	CANADA	32.3	58.5	1454
Estrogen, Diet, Genetics and Endometrial Cancer	EDGE	Case-Control	271	244	USA (NJ)	32.3	60.7	515
Fred Hutchinson Cancer Research Center	FHCRC	Case-Control	55	58	USA (WA)	31.0	60.5	113
Multiethnic Cohort Study	MEC	COHORT	326	659	USA (CA, HI)	28.8	65.5	985
								3067

*Sample size before quality control.

### Genotyping and Quality Control

DNA was extracted at each study site from buffy coat or cheek-cell samples following the manufacturer's protocol and genotyped at the University of Southern California using the Infinium Human Exome BeadChip (Illumina Inc., San Diego, CA) as part of the Stage II replication of the E2C2 GWAS. The BeadChip included 9,232 custom markers, 2,211 of which are specifically relevant to EC, in addition to the 247,870 markers coding primarily for protein-altering variants already included in the BeadChip's default design.

Genotype calling was performed with Illumina GenCall on all samples (n  =  3,067) using the MEC cluster file (16,000 multiethnic samples) for the non-custom markers and autoclustering for the custom markers. Variants were excluded from analyses if call rates were < 90% (n  =  115), the variant was monomorphic (n  =  77,521), the loci had no observed founders and missing all genotypes (n  =  1,962), the variant was an insertion or deletion allele (n  =  117), or the variant deviated from Hardy-Weinberg equilibrium at p-value < 0.0001 in any ethnic group (n  =  248). The final disease trait analysis data set contained 177,139 successfully genotyped variants.

In total, 3,031 out of 3,067 samples were successfully genotyped with call rates ≥ 90%. Of these, we removed 40 duplicate samples (genotype concordance rate > 99.9%) used for assay quality control and 15 samples for other quality control reasons. We conducted principal components analysis (PCA) to identify self-reported ethnicity outliers and infer ancestry with EIGENSOFT v 4.2 [25] using 47,097 custom and non-custom SNPs with genotyping rates > 90% and MAF > 1%. The HapMap phase II (build 37) CEU, YRI, and JPT-CHB samples were used as reference populations. Using the first 5 principal components, we determined 7 individuals that were ethnicity outliers and excluded them from analyses. After further removal of 136 outliers (more than 3.5 standard deviations from the mean) of sample heterozygosity by ethnicity, 2,833 women (1,055 EC cases and 1,778 controls) remained for disease trait analysis.

### Statistical Analysis

#### Single variant association analysis

Single variant analyses were performed overall and stratified by self-reported ethnic group. For each SNP, we estimated odds ratios (OR) and 95% confidence intervals (CI) using unconditional logistic regression, assuming an additive genetic model (0, 1, 2 copies of the minor allele) and adjusting for body mass index (BMI in kg/m^2^), age, study site, plate, and the first 4 principal components to account for population stratification. All single variant analyses were performed using PLINK v 1.07 [26].

#### Gene-based analysis

As an additional method to discover rare variants associated with EC, gene-based testing was performed using SKAT-O [27] over all ethnicities. SKAT-O combines gene-burden tests and SKAT, a SNPset level test for association using kernel machine methods, in special cases for an optimized approach that maximizes power. These analyses were also adjusted for BMI, age, study site, plate and the first 4 principal components. In total, 16,245 genes with at least one variant were tested.

#### Statistical significance

We determined single variant association to reach global significance if the unadjusted p-value was <2.82 × 10^−7^, corresponding to a Bonferroni correction for 177,139 tests. Gene-based associations were considered significant for unadjusted p-values <3.08 × 10^−6^, corresponding to a Bonferroni correction for 16,245 tests.

In accordance to NIH/NCI policy all data will be submitted to the database of Genotypes and Phenotypes (dbGaP, http://www.ncbi.nlm.nih.gov/gap).

## Results

Association analyses included 177,139 successfully genotyped variants with MAF > 0 from a total of 257,102 variants included in the array. Population characteristics of the four participating studies (AHS, EDGE, FHCRC, and MEC) are described in Table 1. Mean age at diagnosis for cases ranged from 58.5 years in AHS to 65.5 years in MEC and mean BMI at diagnosis for cases ranged from 28.8 kg/m^2^ in MEC to 32.3 kg/m^2^ in AHS and EDGE. Of the 3,067 samples genotyped, 2,833 were included in the analysis. There were no differences in age, BMI, and ethnicity between excluded cases and those included in the analysis (results not shown). Of these 2,833 individuals, there were 254 self-reported African-Americans, 347 self-reported Asians, 1,686 self-reported Caucasians, 79 self-reported Hawaiians, 360 self-reported Latinas, and 107 who did not report a specific ethnicity (Table 2).

**Table 2:** Cases and Controls by Reported Ethnicity and Study.

	Alberta		EDGE		FHCRC		MEC	Total	
	Case	Control	Case	Control	Case	Control	Case	Control	Case	Control
Caucasian	446	866	196	177	1	0	0	0	643	1043
Latina	0	0	8	8	17	26	98	203	123	237
Asian	0	0	2	0	0	0	117	228	119	228
African American	0	0	18	8	8	15	68	137	94	160
Hawaiian	0	0	0	0	0	0	26	53	26	53
Unknown	27	40	2	4	21	13	0	0	50	57
Total	473	906	226	197	47	54	309	621	1055	1778

### Variant Distribution among Reported Ethnicities

**Figure 1:**
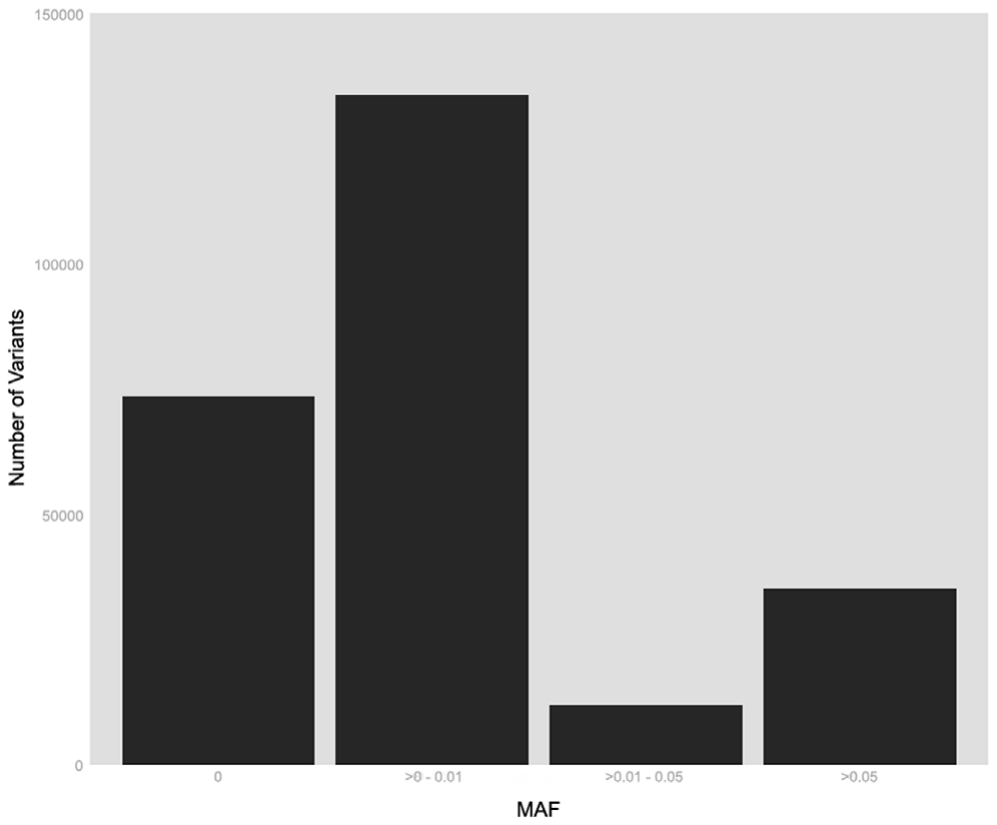
Minor allele frequency for all variants successfully genotyped over all ethnicities. The number of variants is plotted by the minor allele frequency over all ethnicities. These variants include those that are monomorphic in all ethnicities.

**Figure 2:**
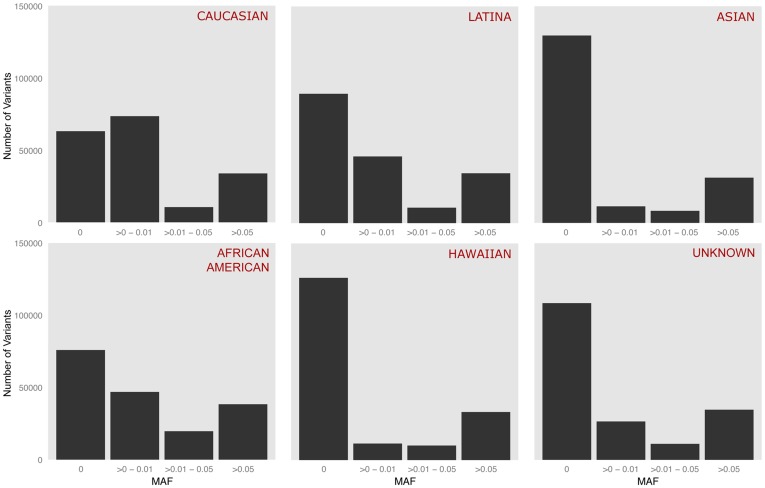
Minor allele frequency for all variants successfully genotyped by reported ethnicity. The number of variants is plotted by the minor allele frequency for each ethnicity. All these variants are polymorphic in at least one reported ethnicity.

**Figure 3:**
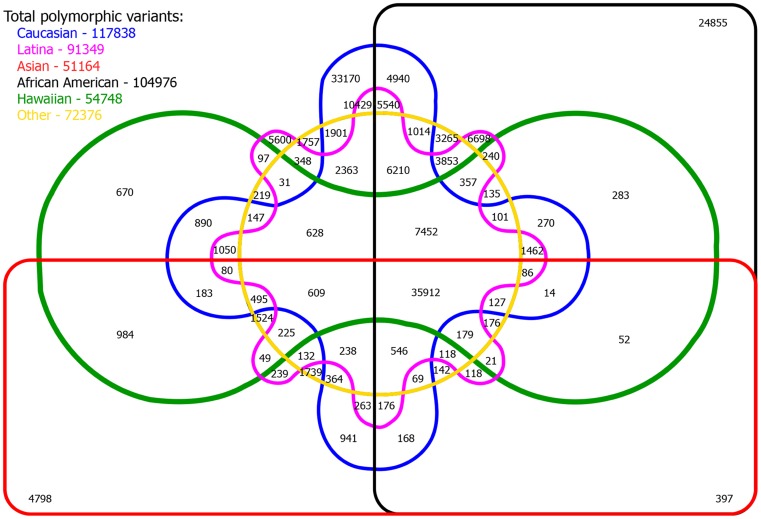
Six-way Venn diagram showing polymorphic putative functional variants shared by reported ethnicities. Numbers of shared variants are shown at intersections. The total numbers of polymorphic variants by ethnicity are listed in the upper-left hand corner.

In this study population, 77,521 variants (30.4%) were found to be monomorphic across all reported ethnicities and 177,139 variants (69.6%) were polymorphic in at least one ethnic population with 74.0% of polymorphic alleles having MAF ≤ 1% (Figure 1). Of the variants that were polymorphic in at least one ethnic population, 42.0% in African Americans, 71.7% in Asians, 34.9% in Caucasians, 69.7% in Hawaiians, 49.5% in Latinas, and 60.0% in those of unknown ethnicity were monomorphic (Figure 2). The MAF distributions were fairly similar among Asians, Hawaiians, and those who did not report a specific ethnicity while African Americans, Caucasians, and Latinas shared more similarities in MAF with each other than with Asians, Hawaiians, and those of unknown ethnicity. About 20.2% (n  =  35,912) of variants were shared by all 5 reported ethnicities while Caucasians and Latinas had the most variants in common at 41.1% (n  =  72,878) (Figure 3). Caucasians had the most unique polymorphic variants (18.7%), followed by African-Americans (14.0%), Latinas (3.2%), Asians (2.7%), those who did not report ethnicity (1.0%), and Hawaiians (0.4%).

### Single Variant Association for Endometrial Cancer

**Figure 4:**
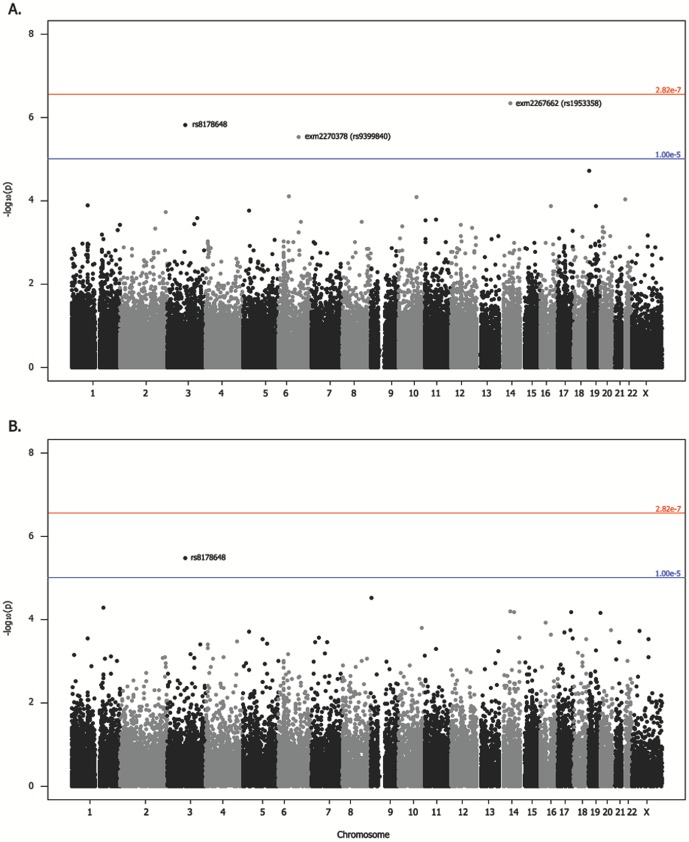
Manhattan plots for the endometrial cancer association analysis. Results of single variant analyses (−log_10_p) are plotted against chromosome position (NCBI build 37) for association over all ethnicities (A) and for associations within Caucasians (B). Suggestive variants are labeled above. Results were adjusted for age at diagnosis, BMI, study site, plate, and the first four principal components.

No variants reached global significance in single variant association of EC for all ethnicities combined (Figure 4a, Table 3) when correcting for multiple comparisons using the Bonferroni adjustment (p <2.82 × 10^−7^). The strongest associations were for variants with >0.05 MAF (Table 3) located within 50 kb of the long non-protein coding intergenic RNA, *LINC00520* (rs1953358, OR  =  1.36, p  =  4.76 × 10^−7^) and in the intron region of *PROS1* (rs8178648, OR  =  1.71, p  =  1.53 × 10^−6^), which codes for protein S, a cofactor to protein C in the anti-coagulation pathway. In Caucasians, who make up the majority of the overall analysis, only rs8178648 remained suggestively associated with OR  =  1.98 and p  =  3.35 × 10^−6^ (Figure 4b, Table 3). There were no globally significant or suggestive variants in African Americans, Asians, Hawaiians, Latinas, and those who did not report ethnicity (Table S1).

**Table 3:** Top five most significant associations of single coding variants with endometrial cancer risk.*

All Cases (n = 1055) vs. Controls (n = 1778)								
Variant	Chr	Position (bp)	Gene/Locus	A1	A2	MAF (all)	OR (95% CI)	P-value
exm2267662 (rs1953358)	14	56295580	LINC00520	G	A	0.49	1.36 (1.20, 1.53)	4.76E-07
rs8178648	3	93605739	PROS1	G	A	0.09	1.71 (1.37, 2.12)	1.53E-06
exm2270378 (rs9399840)	6	104076463	n/a	C	T	0.47	0.75 (0.67, 0.85)	3.01E-06
exm1401784	19	1796166	ATP8B3	T	C	0.23	0.72 (0.61, 0.83)	1.92E-05
exm558041 (rs6926980)	6	56917538	KIAA1586	A	G	0.23	0.75 (0.65, 0.87)	7.95E-05
								
Caucasian Cases (n = 639) vs. Caucasian Controls (n = 1042)								
Variant	Chr	Position (bp)	Gene/Locus	A1	A2	MAF (all)	OR (95% CI)	P-value
rs8178648	3	93605739	PROS1	G	A	0.09	1.98 (1.49, 2.65)	3.35E-06
exm736725 (rs10974657)	9	4622453	SPATA6L	C	T	0.09	2.34 (1.57, 3.50)	3.00E-05
rs10753688	1	165666448	ALDH9A1	C	T	0.41	1.43 (1.20, 1.70)	5.18E-05
exm2267662 (rs1953358)	14	56295580	LINC00520	G	A	0.49	0.71 (0.60 0.84)	6.49E-05
exm1113971 (rs141549345)	14	74401030	LOC283922	A	G	0.03	0.36 (0.22, 0.59)	6.56E-05

*Adjusted for age at diagnosis, BMI at diagnosis, study site, plate, and the first four principal components.

### Gene-based Analysis of Endometrial Cancer

None of the gene-based tests of association were globally significant (p < 3.08 × 10^−6^) after adjusting for multiple comparisons (Table S2). Of the 16,245 genes tested, the most significant EC association was with *KRT81* (p  =  2.21 × 10^−5^), a member of the keratin gene family located on 12q13. *PROS1*, where rs8178648 is located, was not significantly associated with EC (p  =  0.6789) when testing over all ethnicities neither when testing only in Causasians (results not shown).

## Discussion

We present an initial exploration into whether rare variants are associated with EC risk in a multiethnic population from the E2C2. No variants reached global significance (p < 2.82 × 10^−7^) in the single variant association analyses of EC in all ethnicities combined or when stratified by reported ethnicity. Additionally, no gene-based test of association reached global significance (p < 3.08 × 10^−6^).

Among all ethnicities, rs8178648 on chromosome 3 maintained a suggestive association with EC (OR  =  1.707, 95% CI: 1.363–2.123, p  =  1.53 × 10^−6^). The variant lies within the intron region of *PROS1*, a gene coding for protein S, a cofactor in the anticoagulant pathway that causes autosomal dominant hereditary thrombophilia when mutated [28]. *PROS1* expression has been reported to be elevated in aggressive prostate cancer tissue [29] and thyroid cancer tissue [30], suggesting it may have a role in cancer etiology or progression. *PROS1* has been found to be directly upregulated by progestins [31] and downregulated by 17β-Estradiol, an estrogen that regulates gene expression via the estrogen receptor [32], making it susceptible to imbalances in the sex hormone metabolic pathway, which is implicated in EC etiology. However, *PROS1* was not significantly associated with EC (p = 0.6789) when using SKAT-O and no other GWAS have found significant or suggestive variants in this gene.

One weakness of this study is our limited sample size, which was not sufficiently powered to detect rare variants with modest effects associated with EC. Additionally, the exome array content is predominantly based on European ancestry whereas our study included a substantial number of samples with other ancestries. Incomplete exome array coverage of all functional variants and indels that may impact EC risk may also have limited the scope of our study. However, our analysis is one of only two studies [33] using the exome array to examine associations between rare variants and complex diseases in large multiethnic populations. Our study is also the first to utilize the exome array with EC and serves as an extension to our previous examination of common variants on EC risk.

A previous GWAS [13] identified one novel locus near *HNF1B*, rs4430796, inversely associated with EC risk. We replicated the findings in our GWAS [14], but no other common variants associated with EC have been determined. Exome arrays that focus on rare variants, which are hypothesized to have larger effect sizes than common variants, have been used to successfully identify new loci influencing insulin processing and secretion in type 2 diabetics [34]. To date, analyses of cancer sites using exome arrays have failed to find strong evidence that rare variants are highly associated with cancer, revealing only one variant significantly associated with breast cancer and none with prostate cancer [33]. Similarly, we have not identified any loci significantly associated with EC. Due to our limited sample size, our study was estimated to be sufficiently powered to detect ORs > 2.53 for low frequency variants (MAF = 0.02). An OR of 2.00 (MAF = 0.01) would also need around 4,250 cases and 7,250 controls to be sufficiently powered. Even for variants with higher MAFs similar to what was observed for rs8178648, a study detecting a per-allele OR of 1.70 would require at least 1,107 cases and 1,871 controls to be considered sufficiently powered (β = 0.80). Therefore, larger studies need to be conducted in order to detect novel associations with rare variants.

In conclusion, our study found no evidence that rare variants with large effect sizes are associated with EC risk. Though we were able to identify a few suggestive associations, as with rs8178648, much larger studies would be needed to identify a more modest influence of rare variants on the risk of EC.

## Supporting Information

Table S1 **1–5. Single variant association results.** Top 100 most significant single variant associations with endometrial cancer by ethnic groups.
(XLSX)

Table S2 **SKAT-O gene based association results.** SKAT-O gene based associations with endometrial cancer for all ethnicities combined.
(XLSX)
